# Exponential Modeling and Confidence Interval Analysis Reveal Limited Precision of History, Electrocardiogram, Age, Risk Factors, and Troponin (HEART) Score Risk Stratification

**DOI:** 10.7759/cureus.104506

**Published:** 2026-03-01

**Authors:** David R Dobies

**Affiliations:** 1 Interventional Cardiology, National Park Medical Center, Hot Springs, USA

**Keywords:** acute chest pain diagnosis, cardiac risk stratification, clinical decision pathway, heart score, mathematical analysis

## Abstract

Using exponential modeling and confidence interval (CI) analysis, this report evaluates the internal risk behavior of the history, electrocardiogram, age, risk factors, and troponin (HEART) score. The exponential model major adverse cardiac event (MACE %) = 0.79 × e^(0.44·HEART)^ (R²≈0.99) demonstrates substantial CI overlap across HEART 1-3. Only HEART 0-1, combined with non-ischemic ECG and serial negative high-sensitivity troponins, reliably satisfies the American College of Cardiology/American Heart Association (ACC/AHA) <1% 30-day MACE definition. These findings refine the interpretation of intermediate HEART strata and clarify guideline-aligned use.

## Introduction

The history, electrocardiogram, age, risk factors, and troponin (HEART) score was developed heuristically without regression-based optimization. Foundational derivation and validation studies established its clinical use [1-4]. Foundational HEART validations defined major adverse cardiac event (MACE) to include revascularization, a treatment-dependent outcome that may exaggerate differences between HEART score strata [1-4]. Prior HEART implementation and meta-analytic studies have commonly treated HEART scores ≤3 as a unified low-risk category for disposition decisions [5]. The American College of Cardiology/American Heart Association (ACC/AHA) 2021 Chest Pain Guideline defines low risk as an estimated 30-day major adverse cardiac event rate of <1% and recommends structured clinical decision pathways (CDPs) for early discharge of appropriate patients [6]. In contrast, the European Society of Cardiology (ESC) acute coronary syndrome (ACS) guideline (2020, reaffirmed 2023) recommends hs-troponin 0/1-hour or 0/2-hour rule-out algorithms (class I, level B) and uses the Global Registry of Acute Coronary Events (GRACE) score for prognostic stratification and invasive strategy decisions (class I, level A), without including the HEART score among its recommended tools [7].

Although the HEART score is widely applied using fixed cutoffs, it was derived heuristically rather than through regression-based optimization. As a result, adjacent score values may not represent statistically distinct risk strata. This study is a secondary mathematical re-analysis of published summary-level data, designed to evaluate internal score-level precision and interpretive uncertainty rather than to perform a new validation or recalibration of the HEART score.

## Technical report

Objective

To mathematically characterize the history, electrocardiogram, age, risk factors, and troponin (HEART) score's internal risk behavior using exponential modeling and confidence interval (CI) analysis, and to evaluate whether adjacent HEART score values are statistically distinct.

Methods

Published per-score 30-day major adverse cardiac event (MACE) rates and corresponding binomial 95% confidence intervals were extracted from foundational HEART score derivation and validation cohorts [1-3]. The HEART score is a heuristic clinical risk stratification tool developed to estimate short-term adverse cardiac events in patients presenting with acute chest pain [1,2].

Per-score MACE rates were derived from foundational HEART score derivation and validation cohorts, with per-stratum sample sizes ranging from approximately the low hundreds to low thousands, contributing to widening binomial confidence intervals at lower event rates.

Confidence interval overlap was used to assess statistical separability between adjacent HEART score strata, as the objective was to evaluate internal score resolution rather than to perform formal hypothesis testing or equivalence testing between predefined groups.

The American College of Cardiology/American Heart Association (ACC/AHA) 2021 Chest Pain Guideline recommends the use of structured clinical decision pathways to identify patients with an estimated <1% 30-day MACE risk who may be considered low risk and eligible for early discharge (class I, level B-NR) [5]. In contrast, the European Society of Cardiology (ESC) acute coronary syndrome guideline emphasizes high-sensitivity troponin 0/1-hour or 0/2-hour rule-out algorithms and the Global Registry of Acute Coronary Events (GRACE) score for prognostic stratification and invasive strategy decisions, without incorporating the HEART score as a recommended risk tool (class I, level A) [7].

Log-linear regression and exponential curve fitting were performed using De Novo software (De Novo Software, Los Angeles, CA, USA) to estimate the relationship between HEART score values and observed MACE rates across HEART scores 0-7. Confidence interval lower bounds slightly below 0% were truncated to 0.0% for interpretability. Overlap of 95% confidence intervals across adjacent HEART score strata was assessed to evaluate statistical separability rather than formal equivalence or hypothesis testing. Further methodological details, including model derivation, sensitivity analyses, and reproducibility, are provided in the Appendix. 

Results

HEART score 1-3 demonstrated extensive CI overlap, with partial overlap extending through HEART score 4. Scores ≥5 showed rising risk with wider CIs. The fitted exponential model was: MACE (%) = 0.79 × e^(0.44·HEART)^ with R²≈0.99 (Table 1).

**Figure 1:**
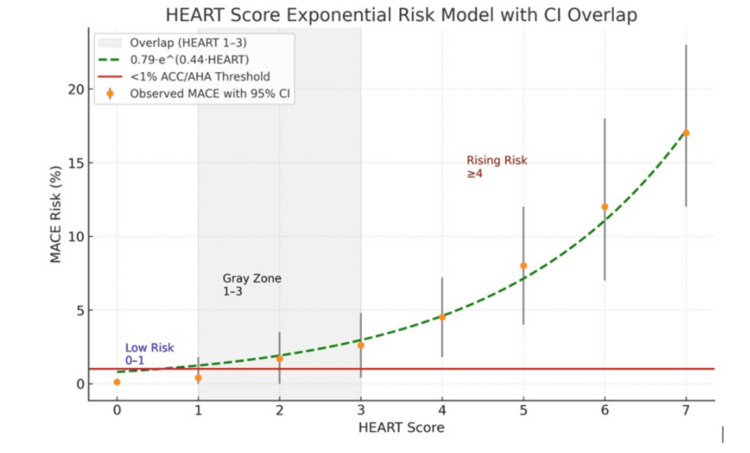
HEART score exponential risk model with confidence interval overlap. Exponential model of estimated 30-day major adverse cardiac event (MACE) risk across HEART score values with 95% confidence interval (CI) overlap. The shaded region highlights HEART scores 1-3, demonstrating substantial overlap and limited statistical separability among adjacent strata. ACC: American College of Cardiology; AHA: American Heart Association; HEART: history, electrocardiogram, age, risk factors, and troponin.

Figure 1 illustrates the fitted exponential relationship between HEART score and estimated 30-day MACE risk, with corresponding 95% confidence intervals used to assess statistical separability across score strata.

**Table 1:** HEART score MACE rates and 95% confidence intervals. Observed 30-day major adverse cardiac event (MACE) rates and corresponding 95% confidence intervals across HEART score strata, derived from foundational validation cohorts. HEART: history, electrocardiogram, age, risk factors, and troponin.

HEART score	MACE (%)	95% Confidence interval
0	0.1	0.0-0.3
1	0.4	0.0-1.8
2	1.7	0.0-3.5
3	2.6	0.4-4.8
4	4.5	1.8-7.2
5	8.0	4.0-12.0
6	12.0	7.0-18.0
7	17.0	12.0-23.0

Clinical implications

HEART 0-1 reliably predicts <1% MACE only when combined with serial hs-troponins and a non-ischemic ECG [5]. HEART 2-3 represent a statistical gray zone and warrant observation and clinical judgment [1-3]. HEART ≥4 reflects rising exponential risk [1-3]. Consistent with ACC/AHA guidance, HEART 0-1 meets the class I low-risk definition, and early discharge aligns with the class IIa recommendation [5]. ESC pathways, in contrast, rely on hs-troponin algorithms and GRACE rather than HEART cut-offs to guide outpatient management [7].

All numerical values were independently extracted from published summary data and reanalyzed; the table and figure were generated by the author using De Novo software and do not reproduce any published table or figure.

## Discussion

Multiple derivation, validation, and implementation studies have historically recommended the HEART score ≤3 as a threshold for low-risk discharge, often treating this range as a unified category [1-3,6]. However, these studies frequently pooled HEART score values 0-3 without examining internal score-level precision and commonly relied on composite MACE definitions that included revascularization, a treatment-dependent outcome that may exaggerate apparent risk differentiation [1-3,4,6]. Additionally, many foundational cohorts predated widespread adoption of high-sensitivity troponin assays, limiting applicability to contemporary chest pain pathways [1-3,6].

In contrast, the present analysis evaluates HEART score strata at the individual score level using exponential modeling and confidence interval overlap to assess statistical separability. This approach demonstrates that HEART scores 1-3 exhibit substantial confidence interval overlap and cannot be assumed to represent distinct or reliably ordered risk categories. Rather than contradicting prior validation studies, these findings refine their interpretation by highlighting the mathematical imprecision within intermediate HEART strata. These findings are consistent with the ACC/AHA emphasis on identifying patients with reliably low (<1%) short-term risk [5] and align with ESC guideline preferences for troponin-driven pathways and GRACE-based prognostic stratification over fixed score cutoffs [7]. Accordingly, these findings describe the internal mathematical precision of the HEART score itself and should not be interpreted as prospective patient-level risk prediction.

## Conclusions

Mathematical modeling reveals that HEART score 1-3 is statistically indistinguishable, whereas only HEART score 0-1 reliably satisfies the ACC/AHA <1% MACE threshold. These findings support using HEART within structured CDPs and reinforce ESC preferences for hs-troponin-based pathways and GRACE over uncalibrated score thresholds. These findings provide a quantitative framework for understanding the internal limitations of score-based risk stratification and underscore the need for caution when interpreting intermediate HEART score values. Incorporating confidence interval behavior into risk assessment may improve bedside decision-making by clarifying which score thresholds are supported by reliable separation versus those that warrant observation and clinical judgment. Future work should explore the integration of such modeling approaches with contemporary high-sensitivity troponin algorithms to further refine chest pain decision pathways.

## Appendices

1. Exponential model derivation: MACE (%) = 0.79·e^(0.44·HEART)^, R²≈0.99.

2. CI analysis: HEART score 1-3 show extensive overlap.

3. Model comparison: Exponential fit outperformed linear and logistic approaches.

4. Sensitivity analyses: Findings remained stable when varying anchoring assumptions.

5. Reproducibility: Model derivation is feasible using publicly available cohort data [1-3].

## References

[1] Backus BE, Six AJ, Kelder JC (2013). A prospective validation of the HEART score for chest pain patients at the emergency department. Int J Cardiol.

[2] Six AJ, Backus BE, Kelder JC (2008). Chest pain in the emergency room: value of the HEART score. Neth Heart J.

[3] Mahler SA, Riley RF, Hiestand BC (2015). The HEART pathway randomized trial: identifying emergency department patients with acute chest pain for early discharge. Circ Cardiovasc Qual Outcomes.

[4] Laureano-Phillips J, Robinson RD, Aryal S (2019). HEART score risk stratification of low-risk chest pain patients in the emergency department: a systematic review and meta-analysis. Ann Emerg Med.

[5] Gulati M, Levy PD, Mukherjee D (2021). 2021 AHA/ACC/ASE/CHEST/SAEM/SCCT/SCMR guideline for the evaluation and diagnosis of chest pain: executive summary: a report of the American College of Cardiology/American Heart Association Joint Committee on Clinical Practice Guidelines. Circulation.

[6] Van Den Berg P, Body R (2018). The HEART score for early rule out of acute coronary syndromes in the emergency department: a systematic review and meta-analysis. Eur Heart J Acute Cardiovasc Care.

[7] Collet JP, Thiele H, Barbato E (2023). 2023 ESC guidelines for the management of acute coronary syndromes. Eur Heart J.

